# Non-human Primate Models to Explore the Adaptive Mechanisms After Stroke

**DOI:** 10.3389/fnsys.2021.760311

**Published:** 2021-11-08

**Authors:** Noriyuki Higo

**Affiliations:** Human Informatics and Interaction Research Institute, National Institute of Advanced Industrial Science and Technology, Tsukuba, Japan

**Keywords:** brain lesions, functional recovery, macaque monkey, marmoset, pain, plasticity, rehabilitation, voluntary movements

## Abstract

The brain has the ability to reconstruct neural structures and functions to compensate for the brain lesions caused by stroke, although it is highly limited in primates including humans. Animal studies in which experimental lesions were induced in the brain have contributed to the current understanding of the neural mechanisms underlying functional recovery. Here, I have highlighted recent advances in non-human primate models using primate species such as macaques and marmosets, most of which have been developed to study the mechanisms underlying the recovery of motor functions after stroke. Cortical lesion models have been used to investigate motor recovery after lesions to the cortical areas involved in movements of specific body parts. Models of a focal stroke at the posterior internal capsule have also been developed to bridge the gap between the knowledge obtained by cortical lesion models and the development of intervention strategies because the severity and outcome of motor deficits depend on the degree of lesions to the region. This review will also introduce other stroke models designed to study the plastic changes associated with development and recovery from cognitive and sensory impairments. Although further validation and careful interpretation are required, considering the differences between non-human primate brains and human brains, studies using brain-lesioned non-human primates offer promise for improving translational outcomes.

## Introduction

The regeneration of lost neural circuitry after stroke, the most frequent cause of brain lesions, is poor because mature neurons do not divide to replace the lost neurons and also because the presence of inhibitory factors prevents functional and structural recovery of the lesioned neuronal tissue (Buchli and Schwab, 2005; Galtrey and Fawcett, 2007). Nevertheless, functional recovery often occurs in patients with stroke and physical and cognitive deficits. In addition to the acute management of stroke to prevent the expansion of irreversible tissue lesions, rehabilitation treatment during the subacute and chronic phases is important to promote functional recovery. Plastic changes of the nervous system and functional compensation in the remaining intact brain areas are thought to underlie the functional recovery after brain lesions, and the concept of neurorehabilitation, which focuses on enhancing plasticity following brain lesions, has received considerable attention over the past decades. However, for the development of effective neurorehabilitation strategies, the functional compensation mechanisms should be elucidated in detail. In addition to clinical studies in stroke patients, studies using animal models in which lesions are experimentally induced in the brain have made major contributions in this research field. Experimental animals can be used to investigate the micro- and macroscopic changes that occur during functional recovery after stroke, including the changes involving gene and protein expression, nervous system structures, and brain activity. Rodents such as mice and rats are the most commonly used vertebrates in biomedical research because of their low cost and ease of genetic manipulation. However, in addition to studies using rodents, investigations using non-human primates such as macaques and marmosets are likely to facilitate translational outcomes because of the proximity of these primates to humans in terms of genetics, anatomy, physiology, and behavior (Kuypers, 1982; Alstermark et al., 2004; Courtine et al., 2007; Isa et al., 2007; Lemon, 2008; Yamamoto et al., 2013; Higo, 2014). Especially, the similarity in the pattern of myelination (Van Essen et al., 2019) is essential because the debris of myelin is produced after stroke and they are toxic to neurons (Marin and Carmichael, 2019). Moreover, recent comparative transcriptomic studies have reported higher genetic similarities in both neurons and microglia among primates as compared to rodents (Geirsdottir et al., 2019; Krienen et al., 2020). This review highlights recent advances in the development of non-human primate models used to explore the adaptive mechanisms after stroke.

## Primate Species

Macaque monkeys, a genus of Old World monkeys that include rhesus monkeys (*Macaca mulatta*), Japanese monkeys (*Macaca fuscata*), and cynomolgus monkeys (*Macaca fascicularis*), are the most commonly used group of primate species in this research field, although other Old World monkeys, such as olive baboons (*Papio anubis*) and vervet monkeys (*Chlorocebus pygerythrus*), have also been used historically (Vilensky and Gilman, 2002). Old World monkeys have a gyrencephalic brain, i.e., brains with a highly folded cortex (Table 1), and cortical and subcortical anatomy similar to that in humans. The neuronal structures of the motor cortex and corticospinal tract of these monkeys are especially more compatible with humans than the other experimental primate species described below (Kuypers, 1982; Alstermark et al., 2004; Courtine et al., 2007; Isa et al., 2007; Lemon, 2008). The combination of this homology of the motor system with the relatively large brains enables acquisition of imaging data on par with those evaluated in clinical research. Macaque monkeys and other Old World monkeys are highly dexterous and capable of precision grip, which is exemplified by the ability to hold small objects between the tips of the index finger and the thumb (Table 1) (Heffner and Masterton, 1975). The common marmoset (*Callithrix jacchus*), a small New World monkey species, has also been used as an animal model. Although common marmosets show a lower degree of dexterity than macaque monkeys, as characterized by the absence of a precision grip (Table 1) (Heffner and Masterton, 1975), their small body size makes them easy to handle. In addition, the brain of common marmosets has a smaller number of cortical sulci, which is referred to as the lissencephalic brain. This characteristic provides advantages over the macaque monkey in experiments such as electrophysiological recording and cortical surface imaging. Moreover, genetic engineering techniques have gradually become applicable to common marmosets (Sasaki et al., 2009). Squirrel monkeys (*Saimiri sciureus*) and capuchin monkeys (*Cebus apella*) are New World monkey species classified as lissencephalic and gyrencephalic primates, respectively (Table 1). Their body sizes are larger than those of common marmosets and smaller than those of macaques. Capuchin monkeys are highly dexterous and use a precision grip, whereas squirrel monkeys have moderately dexterous hands with pseudo-opposable thumbs that can be opposed to the side of the index finger (Heffner and Masterton, 1975). Differences in the extent of corticospinal terminations within the ventral horn are thought to underlie the differences in dexterity between squirrel and capuchin monkeys (Bortoff and Strick, 1993). The species of non-human primates should be selected depending on the purpose of the research. Comparison of the recovery after brain lesions among the different primate species will provide information on how difference in neuronal structures affects the adaptability, which is essential for extrapolating findings obtained by animal studies to human patients. The comparison is, however, difficult at present because the size and location of lesions are highly variable among studies depending on methods of lesion induction as described in the next section. Induction of controlled focal lesions to a specific brain region will enable the comparison among different species.

**TABLE 1:** Non-human primate species used to investigate adaptive mechanisms after stroke.

Species	Body weight (kg)*	Gyrification**	Precision grip
Rhesus monkey	8.3	Gyrencephalic	Capable
Japanese monkey	9.9	Gyrencephalic	Capable
Cynomolgus monkey	4.7	Gyrencephalic	Capable
Olive baboon	25.0	Gyrencephalic	Capable
Vervet monkey	4.0	Gyrencephalic	Capable
Common marmoset	0.4	Lissencephalic	Incapable
Squirrel monkey	0.8	Lissencephalic	Incapable
Capuchin monkey	1.3	Gyrencephalic	Capable

*The information of the average body weight is referred from the articles (Heffner and Masterton, 1975; Bonadio, 2000; Fooden and Aimi, 2005).

**Images of cerebral cortex of the non-human primates can be viewed in the article (Gonzales et al., 2015).

## Methods of Lesion Induction

Since more than 100 years ago, experimental lesions have been made in the motor cortex of macaque monkeys by using various methods such as subpial aspiration, electric thermocautery, and excision with scalpel, and subsequent motor recovery has been investigated (Ogden and Franz, 1917; Travis and Woolsey, 1956; Passingham et al., 1983; Liu and Rouiller, 1999; Vilensky and Gilman, 2002). Because of the presence of a topographically organized map of body parts in the primary motor cortex, which is often identified by intracortical microstimulation techniques (Stoney et al., 1968; Kwan et al., 1978; Higo et al., 2016), it is possible to create a lesion in the cortical areas involved in movements of specific body parts. In recent decades, ibotenic acid, which selectively excites and destroys cell bodies (Curtis et al., 1979), has also been used to create specific gray matter lesions without damaging the white matter (Liu and Rouiller, 1999; Murata et al., 2008, 2015a,b; Yamamoto et al., 2019). However, the pathophysiology of these lesion models may differ from that of stroke patients because the stroke is caused by a blood clot or bleeding, *i.e.*, ischemic and hemorrhagic strokes, respectively. To imitate ischemic stroke, electrocoagulation techniques have been used to occlude the superficial blood vessels supplying the motor cortex (Nudo et al., 1996; Frost et al., 2003; Dancause et al., 2005, 2006). Photothrombotic stroke is another model of superficial blood vessel occlusion (Ikeda et al., 2013; Khateeb et al., 2019). In this method, hydrophilic dyes, such as Rose Bengal, induce platelet aggregation and occlusion of microvessels by light exposure, impairing blood flow within the area exposed to light (Jourdan et al., 1995; Saniabadi et al., 1995). In addition, focal cerebral ischemia was induced in the visual cortex of marmoset through intracortical injections of endothelin-1 (Teo and Bourne, 2014), a vasoconstrictor peptide (Yanagisawa et al., 1988).

**FIGURE 1:**
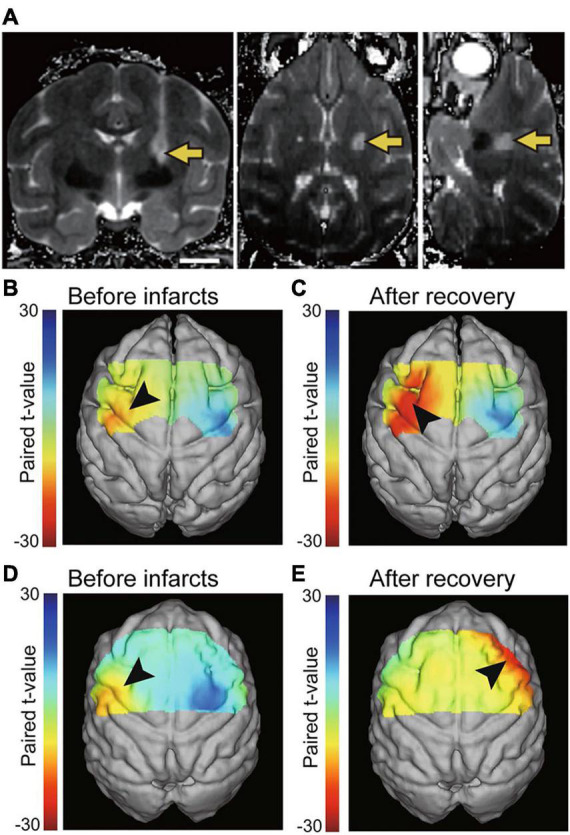
**(A)** T2-weighted MRI showing the location of infarcts in the posterior internal capsule one day after endothelin-1 injection (coronal, axial, and sagittal images). The arrows indicate the infarct. Scale bars = 10 mm. Reproduced from Figure 1 of the study by Murata and Higo (2016). **(B–E)** Brain activation during voluntary hand movements before infarcts **(B,D)** and after motor recovery from the internal capsular infarcts. Before infarcts, focal activation was observed in the hand area of the primary motor cortex (arrowhead in **B,D**). After motor recovery, increased activation of the premotor area was identified (arrowhead in **C,E**). The cortex contralateral to the stroke plays a greater role in recovery when lesions are more severe **(E)**. Reproduced from Figures 2, 4 of the study by Kato et al. (2020b).

Clinically, the middle cerebral artery (MCA) is the most common artery involved in stroke (Nogles and Galuska, 2021). To reproduce the pathology of MCA occlusion, arterial blockade can be induced in the MCA of macaque monkeys by using various methods, including occlusion by a clip or ligature, intra-arterial insertion of filaments or catheters, injection of endothelin-1, or electrocoagulation (Frykholm et al., 2000; Enblad et al., 2001; Virley et al., 2004; Sasaki et al., 2011; Yin et al., 2013; Mcentire et al., 2016; Fan et al., 2017; Yeo et al., 2019). These MCA occlusion models show behavioral and motor function deficits that resemble those seen in stroke patients and are characterized by a relatively large volume of brain lesions, although the volume of lesions differs depending on the nature (permanent or transient), duration, and position of the occlusion. Therefore, MCA occlusion models are associated with a high risk of mortality, and even if the animals do not die, their functional recovery is generally limited because of the severe brain lesions. Although these MCA occlusion models have contributed to the development of strategies for preventing the expansion of irreversible tissue lesions in the acute phase of cerebral infarction, most MCA occlusion models are not ideal for investigating the plastic changes associated with recovery of function by rehabilitation treatments. To investigate the recovery mechanisms after stroke, a smaller focal stroke was induced in the posterior internal capsule, which carries the corticospinal tracts. In comparison with models involving motor cortex lesions, capsular infarct models are expected to be useful for exploring key factors that regulate motor recovery after infarcts in stroke patients because the severity and outcome of motor deficits depend on the degree of lesions to the region (Wenzelburger et al., 2005; Schiemanck et al., 2008; Rosso et al., 2011). In the marmoset capsular infarct model, electrocoagulation was performed in the anterior choroidal artery, which irrigates the internal capsule (Puentes et al., 2015). On the other hand, in the macaque model, focal infarcts were induced at the posterior internal capsule by injection of endothelin-1 (Figure 1A) (Murata and Higo, 2016). Hemorrhage, another major cause of stroke, has also been induced by the injection of collagenase type IV, which disrupts the basal lamina of blood vessels (Rosenberg et al., 1990), into the posterior internal capsule of macaque monkeys.

## Motor Dysfunction and Recovery

Most brain-lesioned non-human primates have been used to study the mechanisms underlying deficits and recovery of motor function after stroke. This is because severe motor deficits are the predominant cause of long-term disability, and motor recovery is the most crucial aspect influencing the quality of life of stroke survivors. Another reason is the experimental ease; objective evaluations of motor performance are more accessible than those of other brain functions. Studies using the motor cortex lesion models described above have indicated recovery of motor performance after paralysis, and therefore the models have been used to investigate mechanisms of motor recovery after lesions to specific brain regions. To date, compensatory changes in brain activity during voluntary movements, movement representations, projections, and gene expression in the motor cortex are reported in such motor cortex lesioned models (Nudo et al., 1996; Liu and Rouiller, 1999; Frost et al., 2003; Dancause et al., 2005, 2006 #17; Murata et al., 2008, 2015a,b; Yamamoto et al., 2019).

A recent study used the macaque model of internal capsular infarcts to report compensatory changes in motor cortical activity during voluntary movements (Figures 1B,C) (Kato et al., 2020b). Similar changes in motor cortical activity have also been reported in stroke patients during motor recovery (Loubinoux et al., 2007; Horn et al., 2016); therefore, the macaque internal capsular infarct model is thought to reproduce the plastic neural changes that occur in stroke patients. Especially, the results of the macaque model of internal capsular infarcts are consistent with stroke patients in that the cortex contralateral to stroke plays a greater role in recovery when lesions are more severe (Figures 1D,E) (Johansen-Berg et al., 2002; Schaechter and Perdue, 2008; Bestmann et al., 2010; Rehme et al., 2011; Bradnam et al., 2012; Kantak et al., 2012; Bajaj et al., 2016; Touvykine et al., 2016; Dodd et al., 2017). Uncovering compensatory mechanisms that occur in the contralesional hemisphere will contribute to understanding hyper-adaptability of the brain, i.e., dynamic reconstruction of the neural structure to compensate for the loss of neural function due to brain lesions. Moreover, brain imaging technologies to monitor brain activity changes in both hemispheres after stroke will be useful to estimate the progress of rehabilitation on functional recovery of stroke patients.

## Cognitive and Sensory Dysfunctions

Stroke patients usually show deficits in multiple functions, including cognitive and sensory functions; therefore, development of relevant animal models that reproduce these deficits is desirable. The anatomical structures and functions involved in cognitive processing in non-human primates are also more similar to those in humans than in rodents. For example, macaques and humans share homologous anatomical and physiological features of the prefrontal cortex, whereas no obvious homologous areas of the prefrontal cortex are found in rodents (Preuss, 1995; Ongur and Price, 2000). The sensory processing system of non-human primates is also more similar to that of humans than those of rodents; for example, the primary somatosensory cortex is well developed in macaque monkeys and clearly differentiated into four subdivisions (areas 3a, 3b, 1, and 2), which are not observed in rodents (Kaas, 2004). Therefore, brain-lesioned non-human primate models designed to study plastic changes associated with the development of and recovery from both cognitive and sensory impairments have also been sought to improve translational outcomes of treating cognitive disorders after physical brain lesions.

In comparison with rodents, non-human primates have a higher potential to perform complex tasks requiring higher cognitive skills, such as planning, decision-making, and problem-solving, and cognitive dysfunction after stroke can be assessed by the performance on cognitive tasks using apparatus such as the touchscreen-equipped operant chamber. To date, cognitive and motor dysfunctions have been assessed in MCA occlusion models with a relatively large volume of brain lesions. One study using an MCA occlusion model in macaque monkeys reported a decline in motor-planning ability for the non-paretic extremity (Roitberg et al., 2003). The marmoset MCA occlusion model also exhibited a neglect of the contralesional side of space (Marshall and Ridley, 2003), which is similar to hemispatial neglect syndrome seen in stroke patients. Hemispatial neglect has also been reproduced in macaque monkeys by inducing reversible lesions of the parietal cortex using the GABA_*A*_ receptor agonist muscimol (Kubanek et al., 2015). These models are important for developing effective neurorehabilitation strategies because hemispatial neglect is a common disabling condition following stroke. However, the studies to date have not addressed the neurological mechanisms underlying both development of and recovery from hemispatial neglect, necessitating further investigation to obtain knowledge for treating cognitive impairment in stroke patients.

**FIGURE 2:**
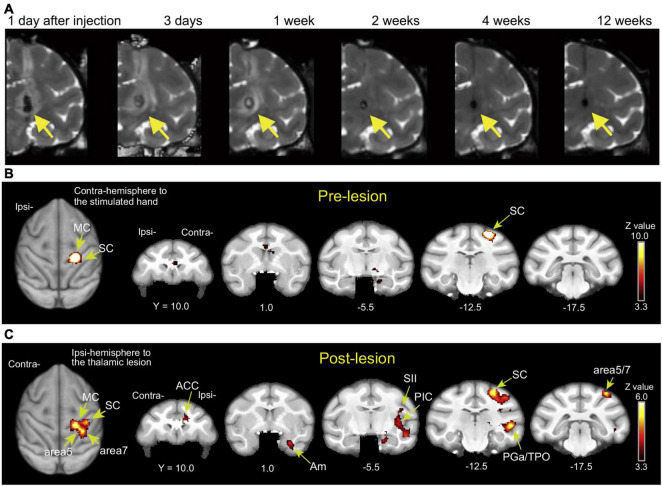
**(A)** T2-weighted MRI showing the time course of hemorrhagic stroke after collagenase type IV induction (coronal images). A hematoma and edema were seen as the hypointense stroke core in the VPL of the thalamus (arrows) and the surrounding hyperintense rim. Reproduced from Figure 1 of the study by Nagasaka et al. (2017). **(B,C)** Brain activity changes associated with CPSP. Brain activity associated with mechanical stimulation to the hand contralateral to the lesioned hemisphere (or the corresponding hand before stroke) during the pre-lesion and post-lesion periods when CPSP was developed. ACC, anterior cingulate cortex; Am, amygdala; MC, primary motor cortex; PGa, anterior subdivisions of the angular gyrus; PIC, posterior insular cortex; SC, primary somatosensory cortex; SII, secondary somatosensory cortex; TPO, temporo-parieto-occipital junction. Reproduced from **Figure 3** of the study by Nagasaka et al. (2020).

Pain is a complex phenomenon that involves sensory, cognitive, and emotional neuronal processing. Pathological pain commonly occurs after stroke and is referred to as central post-stroke pain (CPSP). CPSP occurs over a variable period, usually weeks to months, after stroke in the thalamic nucleus and other areas involved in the somatosensory pathways (Kumar et al., 2009; Hosomi et al., 2015). A dominant theory of the pathophysiology underlying this pain condition is the maladaptive plasticity of the central nervous system, which constitutes a pain-related network. Identification of CPSP-associated plastic changes in the brain can therefore facilitate an understanding of both the pathogenetic mechanism and the therapeutic strategy. In the macaque model of CPSP (Nagasaka et al., 2017), a hemorrhagic stroke lesion was created by injection of collagenase type IV into the ventral posterolateral nucleus (VPL) of the thalamus, which relays somatosensory information (Figure 2A). Behavioral analysis indicated that this model reproduces late-onset allodynia and hyperalgesia similar to those observed in human patients over several weeks and more following the stroke, and both the withdrawal threshold for mechanical stimulation and the withdrawal latency for thermal stimulation on the hand contralateral to the lesioned hemisphere are significantly decreased relative to those before the stroke induction (Nagasaka et al., 2017). A functional MRI study in the macaque CPSP model showed changes in somatosensory stimuli-induced brain activation, which is similar to the findings observed in CPSP patients (Figures 2B,C) (Peyron et al., 1998; Seghier et al., 2005; Ducreux et al., 2006; Ohn et al., 2012; Nagasaka et al., 2020). The brain activation, combined with behavioral results, indicated that the macaque CPSP model faithfully reproduces the pain in CPSP patients. Using the macaque CPSP model, a recent study indicated that a significant reduction in synaptic terminals in pain-related cortical areas is associated with the development of CPSP (Nagasaka et al., 2021). Another recent study using the macaque CPSP model showed that functional connectivity is inappropriately strengthened between the mediodorsal nucleus of the thalamus and the amygdala, which are thought to be involved in the emotional aspects of pain, and that repetitive transcranial magnetic stimulation (rTMS) over the primary motor cortex normalizes this strengthened connectivity (Kadono et al., 2021).

## Concluding Remarks

Investigations of the adaptive mechanisms after stroke using non-human primate models are ongoing, as described above. Further elucidation of these mechanisms may facilitate the development of novel and innovative therapeutic approaches for stroke patients. Non-human primate models are also used to evaluate the clinical efficacy of existing drugs and other therapeutic interventions. Although rodents are the most commonly used vertebrate species in biomedical research, drugs and therapeutics that were proven to be effective in rodent models frequently failed clinical trials (Endres et al., 2008). In addition to the previously described differences in the anatomical structures and functions of the brain between primates and rodents, these models have also shown differences in the post-lesion responses in studies using brain-lesioned animals. For example, the time course of the proliferation of macrophages/microglia after brain lesions as well as their function has been suggested to differ between macaques and rodents (Wang et al., 2013; Nagasaka et al., 2017; Yeo et al., 2019; Yew et al., 2019; Kato et al., 2020a). Moreover, neurogenesis is robustly induced in response to stroke in rodents, whereas neurogenesis after stroke is much lower in non-human primates (Liu et al., 1998; Kee et al., 2001; Tonchev et al., 2003). The time course of macrophage/microglia proliferation as well as the low rates of neurogenesis after brain lesions in non-human primates are consistent with those reported in human stroke patients (Thiel et al., 2010; Huttner et al., 2014). A recent study also reported that responses of astrocytes, another crucial player in the pathogenesis of brain lesions, to stroke differ between rodents and primates (Boghdadi et al., 2020). These differences between rodents and primates may underlie failures in clinical trials.

The results of brain imaging studies, in which activity changes during motor recovery and CPSP development are consistent between macaque stroke models and stroke patients as described above (Loubinoux et al., 2007; Horn et al., 2016; Kato et al., 2020b), may indicate the high translational potential of the primate models. Notably, however, the brains of non-human primates are still different from human brains. For example, MRI tractography analysis showed differences in the frontal network anatomy between humans and non-human primate species, including macaque monkeys (Barrett et al., 2020), and transcriptome analyses have indicated differences in gene expression between human and non-human primate brains (Bakken et al., 2016; He et al., 2017). Although further validation and careful interpretation of these findings are required, studies using brain-lesioned non-human primates have the potential to improve translational outcomes.

## Author Contributions

The author confirms sole responsibility for the conception and preparation of this review.

## Conflict of Interest

The author declares that the research was conducted in the absence of any commercial or financial relationships that could be construed as a potential conflict of interest.

## Publisher’s Note

All claims expressed in this article are solely those of the authors and do not necessarily represent those of their affiliated organizations, or those of the publisher, the editors and the reviewers. Any product that may be evaluated in this article, or claim that may be made by its manufacturer, is not guaranteed or endorsed by the publisher.
